# Targeted Atrial Fibrillation Therapy and Risk Stratification Using Atrial Alternans

**DOI:** 10.3390/jcdd10020036

**Published:** 2023-01-20

**Authors:** Neha Muthavarapu, Anmol Mohan, Sharanya Manga, Palak Sharma, Aditi Kishor Bhanushali, Ashima Yadav, Devanshi Narendra Damani, Pierre Jais, Richard D. Walton, Shivaram P. Arunachalam, Kanchan Kulkarni

**Affiliations:** 1 Department of Cardiovascular Diseases, Mayo Clinic, 200 First Street SW, Rochester, MN 55902, USA; 2 Department of Medicine, Karachi Medical & Dental College, W3V6+27P, Block M North Nazimabad Town, Karachi 74700, Pakistan; 3 Department of Internal Medicine, Texas Tech University Health Sciences Center, El Paso, TX 79905, USA; 4 INSERM, Centre de Recherche Cardio-Thoracique de Bordeaux, University of Bordeaux, U1045, F-33000 Bordeaux, France; 5 IHU Liryc, Electrophysiology and Heart Modeling Institute, Fondation Bordeaux Université, F-33600 Pessac, France; 6 Electrophysiology and Ablation Unit, Bordeaux University Hospital (CHU), F-33600 Pessac, France; 7 Department of Radiology, Mayo Clinic, 200 First Street SW, Rochester, MN 55902, USA; 8 Department of Medicine, Mayo Clinic, 200 First Street SW, Rochester, MN 55902, USA

**Keywords:** atrial alternans, atrial fibrillation, p-waves, arrhythmia risk, ablation, pulmonary vein isolation, substrate mapping

## Abstract

Atrial fibrillation (AF) is the most persistent arrhythmia today, with its prevalence increasing exponentially with the rising age of the population. Particularly at elevated heart rates, a functional abnormality known as cardiac alternans can occur prior to the onset of lethal arrhythmias. Cardiac alternans are a beat-to-beat oscillation of electrical activity and the force of cardiac muscle contraction. Extensive evidence has demonstrated that microvolt T-wave alternans can predict ventricular fibrillation vulnerability and the risk of sudden cardiac death. The majority of our knowledge of the mechanisms of alternans stems from studies of ventricular electrophysiology, although recent studies offer promising evidence of the potential of atrial alternans in predicting the risk of AF. Exciting preclinical and clinical studies have demonstrated a link between atrial alternans and the onset of atrial tachyarrhythmias. Here, we provide a comprehensive review of the clinical utility of atrial alternans in identifying the risk and guiding treatment of AF.

## 1. Introduction

Atrial fibrillation (AF) is the most common cardiac arrhythmia observed today, affecting roughly 33 million people globally [1]. Despite its high prevalence, the pathophysiological mechanisms underlying the development of AF, as well as the biomarkers for evaluating the risk of AF, remain poorly understood [1,2]. While heart failure (HF), cardiomyopathy, and stroke are known co-morbidities of AF and significant risk factors of mortality [1,2], AF alone has been shown to double mortality rates in cardiovascular patients [3].

AF was first shown to be instigated by ectopic triggers located in the atrial sleeve of the pulmonary veins by Haïssaguerre et al. [4]. Radio-frequency catheter ablation, primarily attained through isolation of the pulmonary veins, is a common surgical treatment of AF, and long-term follow-up has shown that the rate of freedom from atrial arrhythmia with a single procedure is 54.1% in paroxysmal AF patients and 41.8% in patients with non-paroxysmal AF [5]. Repeated surgical procedures may increase the success rate to 85–90%, but at the cost of increased hospital overhead and decreased quality of life.

To improve the likelihood of success, multiple theories for identifying triggers and ectopic activity initiating outside the pulmonary veins and instigating AF have been investigated. It is now widely accepted that the development and maintenance of AF require both arrhythmogenic triggers and a suitable substrate [6]. Variations in cellular repolarization time courses (measured as action potential durations or APDs) create substrate heterogeneities conducive to the onset of ectopic triggers, a phenomenon that further amplifies with faster heart rates [7]. These substrate heterogeneities play a significant role in the maintenance and development of AF.

Action potential alternans, a cyclic beat-to-beat alternation in APD, interlinked to cystolic calcium (Ca^2+^) and contraction, have been suggested to play a significant role in the generation of proarrhythmic substrates and facilitate re-entry phenomena that can ultimately lead to sustained AF [8]. Although repolarization alternans in the ventricles have been widely studied [9], with evidence linking the presence of T-wave alternans to the onset of tachyarrhythmias and incidence of sudden cardiac death [9,10], the etiology of alterations in P-wave morphology and duration, or atrial alternans, is relatively uninvestigated. The complexity of the atrial structure in comparison with ventricular tissue adds significant challenges in substrate mapping and, in turn, risk stratification in the atria [9,11]. Yet, promising evidence reported over the past decade suggests that P-wave alternans precede the onset of atrial tachyarrhythmias and may serve as a biomarker for risk stratification and treatment of AF [11,12,13,14].

**Figure 1:**
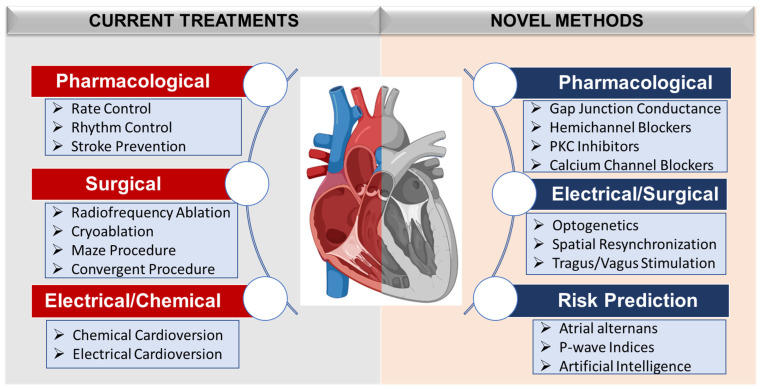
Atrial fibrillation: current and upcoming treatment modalities.

Here, we present a comprehensive overview of the clinical utility of atrial alternans in guiding the treatment of AF by identifying the risk of impending tachyarrhythmia episodes. We summarize the current therapeutic targets for treatment of AF and highlight the existing research gaps (Figure 1). In addition, we review recent preclinical and clinical studies demonstrating the correlation between the presence of P-wave alternans and the onset of AF, along with in-silico studies elucidating mechanistic insights into the origins of localized atrial alternans.

## 2. Investigating Mechanisms of Atrial Alternans

Cardiac alternans, first described by Heinrich Hering in 1908 as a cyclic beat-to-beat alternation in contraction force, intracellular Ca^2+^ release, and APD, is now most commonly believed to occur as a result of the bi-directional association between intracellular Ca^2+^ concentration [Ca^2+^]_i_ and membrane potential (V_m_) [8,15]. V_m_ directly controls the activity of voltage-dependent Ca^2+^ handling systems and [Ca^2+^]_i_ dynamics affect V_m_ regulation via Ca^2+^-dependent ion currents and transporters that define the bi-directional coupling of V_m_ and [Ca^2+^]_i_. Yet, there remains an ambiguity over the fundamental mechanism for the origin of alternans, perturbations of V_m_, or [Ca^2+^]_i_ regulation, as studies have validated both assumptions. Therefore, the mechanisms of alternans remain incompletely understood, especially in atrial tissue.

In the case of atrial tissue, irreversible damage to the atrial substrate over time due to aging causes severe atrial remodeling that can lead to alterations in P-wave duration and morphology [16]. Old age, specifically age >65 years, is considered as one of the most common predictors of AF [17]. In normal healthy atrial myocytes, specific potassium (K^+^) ion channels and their subunits, such as the ultra-rapid delayed rectifier current (I_Kur_) and the transient outward K^+^ current, initiate faster atrial repolarization, leading to shorter APDs compared with ventricular myocytes [11]. However, chronic AF induces APD maladaptation by altering both Ca^2+^ and K^+^ dynamics. AF may lead to reduced mRNA expression for subunits of L-type Ca^2+^ channels, resulting in intracellular Ca^2+^ overload [14], as well as reducing transient outward K^+^ current. These interactions between the ion channel modifications due to aging or ischemia promote shortening of the atrial APD during AF and facilitate atrial APD maladaptation [11,14].

Other factors like intensifying calcium oscillations due to atrial stretch and dilation, remodeling of connexins, and gap junctions also play a role in APD changes [11,18]. These mechanisms lead to APD alternans that acts as a dynamic substrate conducive for the development of AF. It has been postulated that APD alternans at escalating heart rates gives rise to wave front fractionation, wave-break, and conduction block, which instigates the onset of AF [14]. APD alternans was shown to precede every AF initiation episode and rise at slower rates in patients with persistent AF than in those with paroxysmal AF and healthy controls [19]. Furthermore, with continued incremental pacing, APD alternans was shown to transition to complex oscillations at slower rates in persistent AF patients [19].

To ascertain how cellular alterations in patients with AF impacted the manifestations of alternans at heart rates close to rest, electrophysiological remodeling associated with chronic AF was investigated using a computational model of human atrial tissue by Chang et al. [20]. Their results demonstrated that lowering the ryanodine receptor (RyR) inactivation rate reproduced APD alternans observed clinically at slower pacing rates. Decreased RyR inactivation resulted in a steeper sarcoplasmic reticulum (SR) Ca^2+^ release slope, which initiated Ca^2+^-driven alternans. These results highlighted that disturbed Ca^2+^ homeostasis drives proarrhythmic APD alternans in AF patients and may be significantly influenced by RyR kinetics [20], a phenomenon also commonly observed in ventricular tissue [21,22]. Similarly, a recent canine study demonstrated that AF-induced remodeling increased the susceptibility to Ca^2+^ transients and APD alternans by altering calcium dynamics and increasing their variability. Their results indicated that AF remodeling enhanced spontaneous single-cell Ca^2+^ release, prolonged the refractory period of the Ca^2+^ transient, increased the slope of the Ca^2+^ transient restitution curve, and eventually predisposed hearts to Ca^2+^ transient alternans [23]. Furthermore, in a recent simulation study, Zhao et al. [24] revealed that HF-induced atrial electrical remodeling increased Ca^2+^ transient amplitude and SR Ca^2+^ concentration. They demonstrated that the enhanced SR Ca^2+^ reuptake was the primary reason that HF-induced atrial electrical remodeling increased susceptibility to atrial alternans [24].

## 3. Preclinical Models of Atrial Alternans

The role of atrial alternans in AF pathophysiology has been investigated extensively using preclinical animal models over the past couple of decades (Table 1). Atrial remodeling was first demonstrated in 1995 by Wijffels et al. [25], using a goat heart model to elucidate changes in atrial refractory periods and conduction velocities. They hypothesized that AF begets AF, which has been evidenced by numerous animal and human studies in the subsequent years and since widely used as a central principle in the development and perpetuation of AF [25]. A more recent study conducted in ovine hearts confirmed the hypothesis that intermittent in-vivo atrial tachycardia created pro-fibrillatory preconditioning via repolarization alternans and conduction slowing at rapid rates, which in turn created a milieu for the emergence of wave breaks, reentry, and AF [26].

A major risk factor for AF is age and studies conducted in sheep models have shown that aging is associated with a reduction in the threshold for action potential alternans, both in-vivo and in isolated myocytes [27]. The magnitude of APD alternans was observed to increase with age in isolated myocytes and it was reported that age-associated differences in Ca^2+^ handling were responsible for a lower alternans threshold in older animals. It was demonstrated that key changes in calcium dynamics including decreased peak L-type Ca^2+^ current, increased SR Ca^2+^ content, decreased Ca^2+^ transient amplitude, and slowed reuptake of Ca^2+^ into the SR driven by increased Ca^2+^ buffering predisposed the aged myocytes to developing atrial alternans [27]. Their results demonstrated that the alterations in action potential morphology and increased fibrosis slowed conduction of early premature beats in old atria, which is consistent with observations in patients with paroxysmal AF and suggested to contribute to the greater propensity to AF in the aged individuals [28].

Another interesting study tested the correlation between serum K^+^ levels and the risk of AF [29]. In a 12-year prospective study, hypokalemia (<3.5 mmol/L), which has been previously shown to be associated with a higher risk of ventricular arrhythmias and sudden cardiac death, was shown to be indicative of a higher risk of AF [29]. Supporting this theory, a recent study using action potential-clamp experiments demonstrated that APD shortening using K^+^ agonists might be an applicable approach to prevent the development of Ca^2+^ transient alternans in rabbits [30]. Specifically, it was shown that using two specific K+ channel agonists (ML277 for Kv7.1 and NS1643 for Kv11.1) in single atrial myocytes was a promising therapeutic route to suppress the development of alternans, and thus reduce the risk of AF.

## 4. Atrial Alternans and Risk of Atrial Fibrillation

Various features of the P-wave duration and morphology, corresponding to atrial depolarization, have been studied to predict the incidence of AF [12,31,32] (Table 1). Electrical restitution, a fundamental property of cardiac cells that enables the shortening of the APD with an increase in pacing frequencies, has been shown to play a role in instigating tachyarrhythmias at higher heart rates [9,33]. Specifically, alternans are hypothesized to occur when the slope of the restitution curve, the functional relationship between the APD and preceding diastolic interval, exceeds 1 [34,35].

In a clinical study on patients with chronic and paroxysmal AF, inter-regional differences in the slopes of the restitution curves were observed to be greater in patients with chronic AF than in those with paroxysmal AF or healthy controls [31]. It was shown that the spatial nonuniformity of APD restitution characteristics throughout the atrium forms a substrate conducive to the perpetuation of AF [31]. In another study investigating APD kinetics in patients with atrial flutter, 20 out of 38 patients were observed to develop AF at a mean pacing cycle length of 184 ± 38 ms, which was preceded by monophasic action potential alternans at a mean cycle length of 219 ± 45 ms [14]. The study demonstrated that atrial flutter originating at the isthmus initially instigates APD alternans and conduction block, and then progresses to AF.

Another recent case study reported the presence of pulsus alternans, an alternation in the pulse strength that has been linked to severe HF, instigated by atrial flutter, which ceased on cardioversion [36]. A more direct correlation between atrial alternans and AF was reported by a clinical study, investigating the role of calcium dynamics in AF [37]. They demonstrated that discordant alternans was induced by rapid atrial pacing in 68% (13/19) of the enrolled AF patients, and AF was initiated after the induction of discordant alternans in 8/13 of these patients [37]. Furthermore, the anti-arrhythmic drug verapamil was shown to suppress the inducibility of discordant alternans, and thus reduce susceptibility to AF [37].

## 5. Current Therapeutic Targets and Research Gaps

The current pharmacological treatments for AF are mainly divided into three categories: rate control, rhythm control, and stroke prevention. The target audience for rate control therapy are patients aged >65 years, as these medications help reduce recurring hospitalizations by aiming to maintain a resting heart rate of <80 bpm [38]. Commonly used drugs to control heart rate are β blockers and non-dihydropyridine Ca^2+^ channel blockers, digitalis, and amiodarone. For rhythm control, the main goal is to sustain sinus rhythm and attenuate uncomfortable symptoms to improve the everyday quality of life [39].

Direct current cardioversion is an effective therapy for AF patients or AF with rapid ventricular response to restore sinus rhythm, and it is mainly recommended for AF patients who do not respond to pharmacological therapies, combined with HF or hemodynamic instability [5]. In addition, radio frequency catheter ablation is highly recommended for symptomatic paroxysmal AF patients, aiming to prevent recurrent AF and improve symptoms, especially when antiarrhythmic drug therapy is unsuccessful. Cryoablation is also currently used in practice for patients with paroxysmal AF, but the option is only designed for dissection of the pulmonary artery [40].

Pulsed field ablation (PFA) is a promising treatment strategy wherein fast and short-duration non-thermal energy is delivered to cardiomyocytes using electroporation, which damages their structural integrity, leading to isolated death of the target cell. In comparison with other thermal ablation procedures, PFA can be delivered ultra-rapidly in sub-seconds by both percutaneously delivered endocardial and surgically placed epicardial catheters [41]. Thanks to its tissue specificity, it prevents the esophagus, phrenic nerve, and any other surrounding atrial tissue damage [42].

Another surgical option is Cox maze IV procedure, which is currently the gold-standard surgical treatment for AF, with a 93% non-recurrence of AF at one year and a 78% freedom from AF at five years [43]. Finally, the convergent procedure is a two-phase surgical treatment, with the first conducted by a cardiothoracic surgeon who uses a radiofrequency catheter to perform invasive epicardial ablation, and the second by an electrophysiologist who fills in any gaps identified from an intracardiac approach.

In interventional procedures, a novel study investigated the termination of spiral wave arrhythmias using light waves, given its minimal side effects and non-invasiveness [44]. Using optogenetic engineering on the substrate itself, they demonstrated the successful termination of spiral waves in neonatal rat atrial myocytes [44]. Another promising study using a custom monitoring device called MAX-SRS is currently in clinical trials to sense and identify the atrial rhythm and initiate spatial resynchronization therapy upon detection of AF [45]. Finally, a recent non-invasive study investigated the utility of chronic (1 h daily for 6 months) transcutaneous vagus nerve stimulation (tragus stimulation) in AF patients and demonstrated that parasympathetic stimulation led to 85% lower AF burden in patients with paroxysmal AF [46].

## 6. Future Scope: Can Atrial Alternans Guide Atrial Fibrillation Therapy and Risk Stratification?

The search for substrate-driven AF assessment and treatment has been gaining increased research attention over the past decade. Variations in P-wave morphology and duration have been directly linked to the substrate dependent conduction abnormalities observed in AF patients [47]. A novel preclinical study demonstrated that His-electrogram alternans enabled the visualization of dual-pathway electrophysiology by confirming the presence of both slow and fast pathway wavefronts during AF in rabbit atrio-ventricular nodal experiments [48]. Furthermore, variability in P-wave morphology was shown to be effective in predicting the outcome of circumferential pulmonary vein isolation in patients with recurrent AF [16].

Recent clinical studies have also demonstrated the potential of cardiac alternans as a biomarker of vagal stimulation treatment efficacy in patients with both atrial and ventricular tachyarrhythmias [12,49]. Cardiac alternans was shown to be modulated by both acute and chronic changes in electrophysiological substrate by vagus nerve stimulation. In addition, P-wave alternans was demonstrated to have the potential to identify patients likely to benefit from long-term treatment based on the acute changes observed in alternans levels in response to vagal stimulation [12]. These studies demonstrate a direct association between atrial alternans and the incidence of tachyarrhythmias, highlighting its potential for risk stratification in patients with AF.

Despite the promising preliminary results highlighting the utility of cardiac alternans in assessing the risk of AF, further studies are warranted to test its efficacy in substrate mapping for guiding ablation therapy. Extensive evidence demonstrating the predictive capabilities of both T-wave repolarization and P-wave atrial alternans has been reported over the past two decades. Yet, its clinical applicability in guiding treatment strategies is limited, primarily because of the patient-to-patient variability and a non-standardized approach for calculating alternans levels, and hence the impending risk of tachyarrhythmias. Recent data suggest a more patient-specific approach would likely be more effective in guiding treatment strategies. Screening for atrial alternans may provide an indication of the risk of AF and help improve patient management strategies.

**Table 1:** Summary of in-silico, in-vitro, preclinical, and clinical studies investigating the utility of p-wave alternans in predicting AF. HF = heart failure, APD = action potential duration, AF = atrial fibrillation, RyR = ryanodine receptor, SR = sarcoplasmic reticulum.

Authors	Year	Objectives	Model	Primary Results
**IN-SILICO AND IN-VITRO STUDIES**				
Zhao et al. [24]	2020	Investigate the effects of HF-induced electrophysiological and structural remodeling on atrial alternans	Canine atrial 1D model	HF-induced atrial electrical remodeling increased Ca^2+^ transient amplitude and SR Ca^2+^ concentration, resulting in increased susceptibility to alternans
Kanaporis et al. [30]	2019	Can K^+^ agonists prevent the development of Ca^2+^ transient alternans	Rabbit atrial myocytes	K^+^ channel agonists can suppress the development of atrial alternans
Kanaporis et al. [50]	2016	Investigate mechanisms of atrial alternans	Rabbit atrial myocytes	Calcium-activated chloride current determines action potential morphology during calcium alternans
Chang et al. [20]	2014	Determine how cellular remodeling in atrial fibrillation patients affects alternans	Human atrial tissue model	Disturbed Ca^2+^ homeostasis drives proarrhythmic APD alternans in AF patients and may be significantly influenced by RyR kinetics
Tsai et al. [51]	2011	Investigate the effect of mechanical stretch on APD and calcium transient alternans	Atrial myocytes monolayer	The threshold of the APD alternans and calcium transient alternans is significantly reduced
Kockskämper et al. [52]	2002	Study the subcellular properties of Ca^2+^ alternans in atrial myocytes	Feline atrial myocytes	Focal inhibition of glycolysis mechanisms within a myocyte led to a rise in alternating Ca^2+^ waves
Hüser et al. [53]	2000	Understand the mechanisms of alternans by evaluating the SR Ca^2+^ triggers by restricting different glycolysis routes	Feline atrial myocytes	Cardiac alternans result from alterations in the gain of excitation contraction coupling, which is locally controlled around the SR Ca^2+^ release sites by mechanisms utilizing ATP, produced by glycolytic enzymes
**PRECLINICAL STUDIES**				
Liu et al. [23]	2020	Can AF-induced remodeling increase the susceptibility to Ca^2+^ transients and APD alternans	Canine tachypacing model	AF remodeling enhanced spontaneous Ca^2+^ release, prolonged the refractory period of the Ca^2+^ transient, increased the slope of the Ca^2+^ transient restitution curve, and predisposed the hearts to Ca^2+^ alternans
Pearman et al. [27]	2018	Can age-associated differences in Ca^2+^ handling predispose animals to a lower threshold atrial alternans	In vivo ovine model	Decreased Ca^2+^ transient amplitude and slowed reuptake of Ca^2+^ into the SR driven by increased Ca^2+^ buffering predisposed the aged myocytes to develop atrial alternans
Monigatti-Tenkorang et al. [26]	2014	Investigate the mechanisms by which intermittent atrial tachycardia promotes sustained AF	In vivo ovine model	Atrial tachycardia causes a pro-fibrillatory substrate and increased atrial repolarization alternans, leading to AF
Gan et al. [28]	2013	Investigate whether the L-type Ca^2+^ current alters with age, predisposing the heart to AF	Canine model	Aged cells had longer APD and lower peak L-type Ca^2+^ current densities, leading to slow and discontinuous conduction of the left atria
David et al. [32]	1981	Demonstrate mechanical atrial alternans during programmed atrioventricular pacing with and without A-V block	In vivo canine model	Atrial alternans was demonstrated during rapid atrial stimulation at cycle lengths ranging from 250 to 120 ms
**CLINICAL STUDIES**				
Kulkarni et al. [12]	2021	Investigate if p-wave alternans is a marker for utility of low-level tragus stimulation in paroxysmal AF patients	Paroxysmal AF patients	Chronic tragus stimulation significantly reduced p-wave alternans in AF patients and atrial alternans helped identify patients likely to benefit from the treatment
Yoshikawa et al. [36]	2020	Pulsus alternans during atrial flutter	Case study	Presence of pulsus alternans, an alternation in the pulse strength, was observed to be instigated by atrial flutter, which ceased on cardioversion
Siniorakis et al. [13]	2017	P-wave alternans and atrial flutter	Case study	Repeated episodes of atrial flutter were observed, always preceded by p-wave alternans
Lalani et al. [18]	2013	Investigate utility of spectral analysis of alternans as a clinical index of propensity to AF	AF patients	Spectral alternans analysis can identify patients with predisposition to AF
Narayan et al. [19]	2011	Investigate if atrial APD alternans reveals vulnerability to AF	AF patients	APD alternans preceded every AF initiation episode and revealed susceptibility to AF
Hiromoto et al. [37]	2005	Investigate the role of atrial alternans in AF	Patients with structural heart disease	Rapid atrial pacing induced discordant alternans, which was associated with initiation of AF
Kim et al. [31]	2002	Study APD restitution kinetics in AF patients	AF patients	AF was related to steeply sloped (>1) APD restitution kinetics and heterogeneity of APD of the atrium played an important role in the persistence of AF
Narayan et al. [14]	2002	Test the hypothesis that atrial flutter can progress to AF via atrial action potential alternans	Atrial flutter patients	Atrial flutter originating at the isthmus initially instigates APD alternans and conduction block, and then progresses to AF

## Data Availability

Not applicable.

## Abbreviation

AF	Atrial fibrillation
HF	Heart failure
APD	Action potential duration
RyR	Ryanodine receptor
SR	Sarcoplasmic reticulum
V_m_	Membrane potential
Ca^2+^	Calcium
K^+^	Potassium
PFA	Pulsed field ablation
